# Interfacial modification of NaCoO_2_ positive electrodes with inorganic oxides by simple mixing and the effects on all-solid-state Na batteries†

**DOI:** 10.1039/d4ra02957g

**Published:** 2024-06-19

**Authors:** Takaaki Ichikawa, Koji Hiraoka, Shiro Seki

**Affiliations:** a Graduate School of Applied Chemistry and Chemical Engineering, Kogakuin University 2665-1 Nakano-machi Hachioji Tokyo 192-0015 Japan shiro-seki@cc.kogakuin.ac.jp +81-42-628-4568 +81-42-628-4568

## Abstract

All-solid-state Na polymer batteries are desired as the next generation of high-capacity batteries owing to their high safety and abundant resources. However, the degradation of the positive electrode/electrolyte interface with cycling leads to a decrease in capacity and a significant increase in interfacial resistance. In this study, to suppress the interfacial degradation, we prepared positive electrode sheets through a combination of simple mixing and pasting with the addition of binders and conductive additives, using NaCoO_2_ coated with two types of inorganic oxides as the active material. The influence of the coatings on the electrochemical properties of the fabricated all-solid-state Na polymer battery was investigated by performing constant-current charge–discharge tests, and the coating morphology was characterized by electron microscopy and spectroscopic measurements. Compared with the non-coated positive electrode, the coated electrodes not only enhanced the battery capacity and improved the cycling characteristics but also effectively suppressed the formation of byproducts during charge–discharge cycling, owing to the electrochemical stability and Na^+^ conductivity of the inorganic oxide coatings. Moreover, despite the chemically unstable properties of powdered NaCoO_2_, the application of this mixing method effectively suppressed its degradation.

## Abstract 2

All-solid-state Na batteries were prepared using PEO-based electrolytes, and NaCoO_2_ (NCO) was coated with oxide-based materials through simple mixing to protect the NCO surfaces.

## Graphical abstract

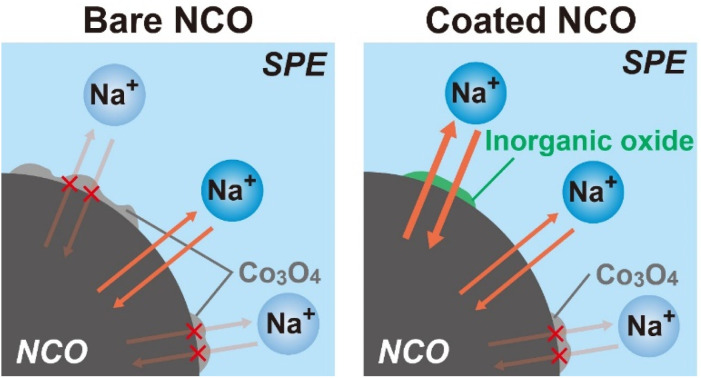

## Introduction

Li-ion batteries have been used in a wide range of applications, from consumer devices, such as smartphones and PCs, to large applications, such as power sources for electric vehicles and primary storage of renewable energy, and the demand for these battery systems is expected to continue expanding in the future. However, Li compounds are relatively rare and unevenly distributed in mineable areas, with a crustal abundance of approximately 20 ppm. There are concerns regarding the price increase in the short term and ensuring a stable supply in the long term. Therefore, it is necessary to develop next-generation secondary battery systems using elements with higher abundance than Li as the reactive ion species.^1–3^ In particular, the elemental abundance of Na in the earth's crust is approximately 1000 times greater than that of Li, and its wide distribution is attributed to its recoverability from seawater.^4^ Therefore, Na batteries are being actively developed as post-Li-ion secondary batteries.^3,5,6^

Moreover, conventional Li-ion batteries comprise flammable organic solvents,^7–9^ posing risks of ignition because of their potential for leakage and thermal runaway properties. To enhance safety, there has been increasing interest in all-solid-state batteries that leverage non-flammable and non-volatile solid electrolytes, such as sulfide, oxide, and organic polymer materials, as alternatives to organic liquid electrolytes.^10–17^ In addition, all-solid-state batteries can be stacked within a single external package without the risk of liquid short-circuiting. This approach is effective for improving both energy density and safety properties. Solid sulfide electrolytes that exhibit Na^+^ conductivity, such as Na_3_PS_4_ and Na_3_SbS_4_, have been reported.^18–20^ These materials exhibit a relatively high order of ionic conductivity, approximately 10^−3^ S m^−1^ at room temperature, and possess a soft amorphous structure that facilitates easy processing. However, their reactivity with moisture in the air is a safety concern, leading to the generation of harmful hydrogen sulfide. In contrast, oxide-based Na-conductive solid electrolytes have been studied, such as β-Al_2_O_3_ with a layered structure and the Na super-ionic conductor type Na_3_Zr_2_Si_2_PO_12_.^21–24^ These materials exhibit favorable chemical stability and safety, despite the difficulty in forming stable interfaces with the electrode owing to their rigid and fragile properties. Alternatively, solid polymer electrolytes (SPEs) exhibit a lower ionic conductivity (approximately 10^−5^ S m^−1^ at room temperature) compared with sulfide and oxide-based solid electrolytes, but SPEs easily form stable interfaces with electrodes owing to their high flexibility and moldability. The rubbery property of SPEs enables the fabrication of flexible all-solid-state batteries by roll-to-roll molding, achieved by laminating electrode/electrolyte sheets. This flexibility also facilitates the adaption of existing Li-ion battery production equipment in the mass-production process. Generally, in SPEs consisting of poly(ethylene oxide) (PEO), metal cations are solvated with ether oxygen ligands. They are cooperatively transported with the segmental motion of the host polymer, resulting in ionic conductivity, and this conduction mechanism is referred to as a coupling-type solid electrolyte.^25^ Na-conductive SPEs can also be fabricated by dissolving Na salt as a cation source into a host polymer, and PEO-based SPEs exhibit higher reductive stability, making them more suitable for combining with metallic-Na negative electrodes,^22,26^ compared with carbonate-based SPEs and others. However, PEO-based SPEs are considered to have relatively low oxidative resistivity. All-solid-state batteries using PEO-based SPE generally degrade during charging process, as a result of oxidative decomposition at the positive electrode/electrolyte interface and irreversible reactions involving the positive electrode active material. Significant degradation of the positive electrode/electrolyte interface, accompanied by an increase in resistance with the charging cycles, has previously been reported in all-solid-state Li-based polymer batteries using LiCoO_2_ (LCO) as the positive electrode active material and PEO-based SPE.^27^ To prevent the degradation at the interface of the positive electrode active material, various methods have been proposed. For example, in liquid-type Li-ion batteries, the surfaces of the positive electrode active material particles have been coated using oxide materials (*e.g.*, Al_2_O_3_ and ZrO_2_), suppressing the capacity degradation with cycling.^28–30^ Nonetheless, there are concerns regarding the complexity and costs of the manufacturing process. In contrast, the protection of LCO particles through simple mixing with highly chemically stable materials, such as Li_3_PO_4_, is effective in suppressing the increase in interfacial resistance and capacity degradation with cycling.^31^ This approach is also expected an easy method of protecting the positive electrode interface. Therefore, in this study, all-solid-state Na polymer batteries (ASSBs) using layered oxide and PEO-based SPEs as active material and electrolyte respectively, are also expected to exhibiting suitable degradation suppression behavior at the positive electrode/SPE interface. Furthermore, if this method can be applied to ASSBs using layered oxides and PEO-based SPEs as the active materials and electrolytes, it is expected to not only suppress capacity degradation derived from the positive electrode/SPE interface degradation with cycling but also enable a broad selection of positive electrode active materials, including moisture-free electrodes such as NaMnO_2_ and Na_0.76_Ni_0.38_Mn_0.62_O_2_.^32,33^

In this study, ASSBs were prepared using PEO-based SPEs as electrolytes, and NaCoO_2_ (NCO) was coated with two oxide-based materials (Na_3_PO_4_ or Na_3_Zr_2_Si_2_PO_12_) through simple mixing to protect the positive electrode active material surfaces. Electrochemical analysis was performed to investigate the effects of the coating on battery performance and degradation behavior with cycling. The proposed concept for interfacial protection was actually demonstrated by structural and surface evaluation using electron microscopy and spectroscopy. This evaluation was performed on the positive electrode sheet before and after cycles, as well as NCO powders, aiming to investigate the detailed degradation suppression effect achieved by the coating of oxide-based materials.

## Experimental

### Preparation of [Na|SPE|NCO] cells and the charge–discharge test

**Fig. 1:**
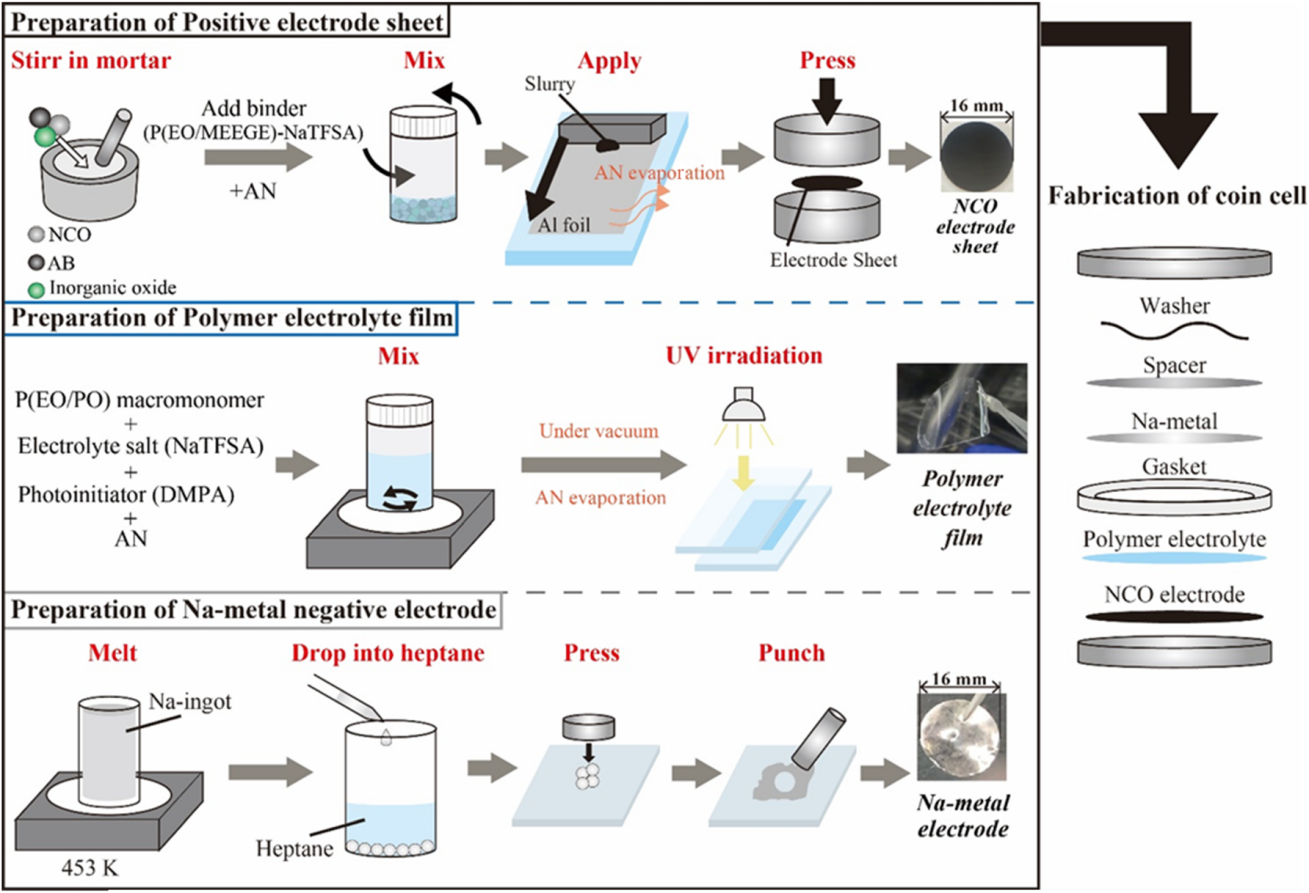
Preparation scheme of each component and the electrochemical cells used for all-solid-state Na-based polymer batteries.

In this study, all fabrication processes of the electrode, electrolyte, and cell were conducted in an Ar-filled glove box (Miwa Manufacturing Co., Ltd, [H_2_O] < 0.5 ppm, [O_2_] < 10 ppm) owing to their instability of NCO in the atmosphere. The inorganic oxides Na_3_PO_4_ (Toshima Manufacturing Co., Ltd) and Na_3_Zr_2_Si_2_PO_12_ (NZSP, Toshima Manufacturing Co., Ltd) were mixed with NCO in a mortar at ratios of 5 wt% (Na_3_PO_4_) and 10 wt% (Na_3_PO_4_ and NZSP) to NCO until these powder materials became uniformly dispersed (see Fig. S1†). The composite positive electrode sheets were prepared by mixing NCO (coated or non-coated) with a conductive additive namely acetylene black (AB, DENKA BLACK Li-250, Denka), and a binder polymer at a ratio of 82 : 5 : 13 by weight (including inorganic oxides for active material). The binder polymer was prepared by dissolving poly(ethylene oxide)-*co*-2-(2-methoxyethoxy)ethyl glycidyl ether (P(EO/MEEGE), Osaka Soda Co., Ltd) and NaN(SO_2_CF_3_) (NaTFSA, Kishida Chemical Co., Ltd) as a Na salt in acetonitrile (AN, Fujifilm Wako Pure Chemicals Co., Ltd) with a concentration of [Na]/[O] = 0.10. The obtained uniform slurry was applied onto the Al foil current collector with a thickness of 50 μm, and then the sheet was punched to *ϕ* 16 mm using a hand punch (Trimming cutter C4, Wista Co., Ltd) and pressed using a uniaxial press to obtain composite positive electrode sheets. SPEs were prepared using a polyethylene oxide/polypropylene oxide random copolymer (P(EO/PO), EO : PO = *ca.* 8 : 2, *M*_w_ = *ca.* 8000, Daiichi Kogyo Seiyaku Co., Ltd) as the macromonomer, NaTFSA as the Na salt, and 2,2-dimethoxy-2-phenyl acetophenone (DMPA, Chiba Japan Co., Ltd) as the photoinitiator. P(EO/PO), NaTFSA, and DMPA (0.1 wt% based on the macromonomer weight) were dissolved in AN with a salt concentration of [Na]/[O] = 0.10. After the electrolyte mixture was vacuum dried for 24 h, the obtained homogeneous solution was cast between two glass plates separated by a poly(tetrafluoroethylene) spacer (Teflon™, 100 μm thickness) and irradiated with UV light for 5 min to initiate crosslinking of the macromonomers, resulting in the SPE films. Metallic-Na negative electrodes were prepared using Na ingots (Sigma-Aldrich). The Na ingot was melted at 453 K, and the liquid-state Na was dropped into heptane (Fujifilm Wako Pure Chemicals Co., Ltd) to obtain spherical Na metal, which were pressed and spread into thin foils. ASSBs (*i.e.*, [Na|SPE|NCO] cells) were assembled using 2032-type coin cells, comprising the composite positive electrode sheet, SPE film, and metallic-Na negative electrode (Fig. 1). The prepared all-solid-state Na cells were annealed at 363 K for 48 h to form a stable electrode/electrolyte interface, and the battery performance was evaluated by performing constant-current charge–discharge tests (HJ1010mSM8A, Hokuto Denko Co., Ltd) in the voltage range of 3.5–2.0 V, with a C/20 rate, at 333 K.

### Surface and structural analysis of the electrode sheets

To examine the morphology of the inorganic oxide coatings on the NCO surfaces such as particle shapes and elemental distributions, scanning electron microscopy/energy dispersive X-ray spectroscopy (SEM-EDX, JCM-6000, JEOL) was performed for the NCO, 10 wt% Na_3_PO_4_-coated NCO, and 10 wt% NZSP-coated NCO powders. To investigate degradation behavior of prepared all-solid-state Na batteries with cycle number by comparing the crystal structure of NCO in the positive electrode sheet, X-ray diffraction (XRD, CuKα = 0.15418 nm, MiniFlex600, Rigaku) was performed at room temperature. After 5 charge–discharge cycles, the [Na|SPE|NCO] cells were disassembled in a dry box ([H_2_O] < 2.6 ppm, DPU02A-SP, ORION), and the positive electrode sheet was peeled off from the SPE film/metallic-Na negative electrode to obtain samples without moisture. The obtained positive electrode sheet was then encapsulated in a sealed cell that enabled X-ray transmission, and XRD measurements were performed in the range of 10–70° at a scan rate of 10° min^−1^.

To evaluate the intrinsic chemical stability of NCO related to degradation of all-solid-state Na cells, Raman spectroscopy was performed at room temperature to observe the changes of bonding state and crystal structure under various environmental conditions (NRS-4500, Jasco). Raman spectra of bare NCO, Co_3_O_4_ (Kojundo Chemical Laboratory Co., Ltd), Na_3_PO_4_- and NZSP-coated NCO powders, and composite positive electrode sheets containing 10 wt% NZSP-coated NCO were measured. The Raman spectra were obtained using a laser wavelength of 532 nm and a power of 15.5 mW, and the optical resolution was calculated to be approximately 4.9 cm^−1^ using a 900 mm^−1^ grating, *ϕ* 34 mm pinhole slit, and 50× objective lens. To evaluate the chemical stability of NCO against moisture and oxygen, Raman spectroscopy was also conducted on positive electrode sheets containing bare NCO powder that were exposed to the atmosphere at room temperature. For all Raman spectra, the wavenumbers of *x*-axis were corrected based on the peak position of the standard sample, namely polypropylene.

## Results and discussion

### Performance of the [Na|SPE|NaCoO_2_] cells

**Fig. 2:**
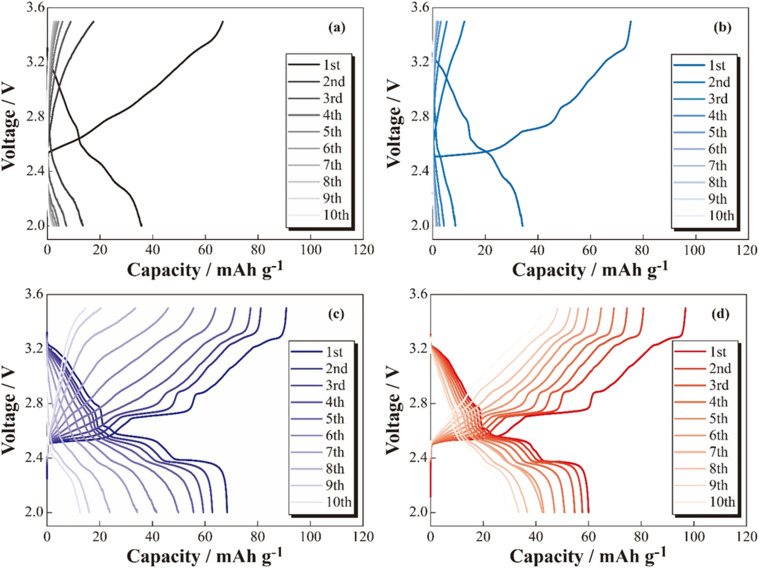
Charge–discharge profiles for the [Na|SPE|NCO] (a), [Na|SPE|5 wt% Na_3_PO_4_-coated NCO] (b), [Na|SPE|10 wt% Na_3_PO_4_-coated NCO] (c) and [Na|SPE|10 wt% NZSP-coated NCO] (d) cells.

**Fig. 3:**
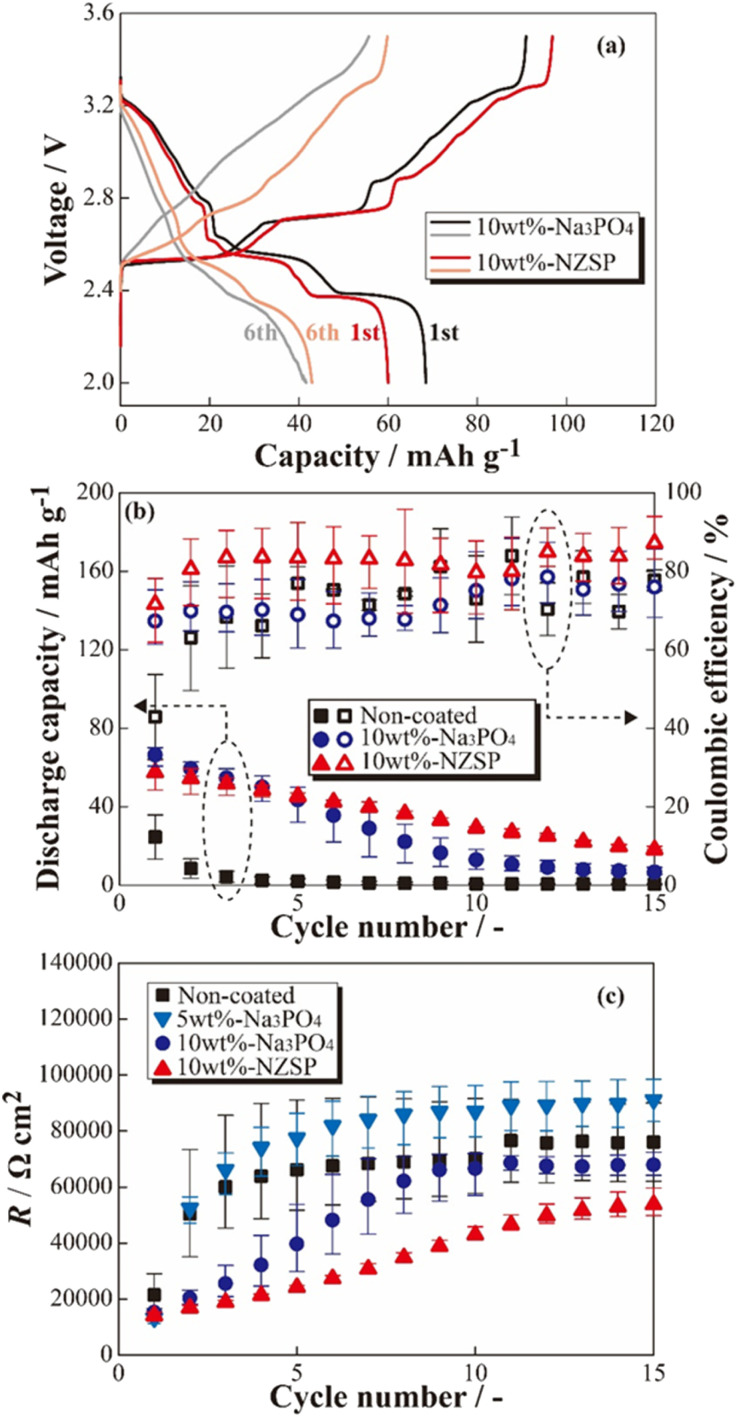
Charge–discharge profiles for the [Na|SPE|10 wt% Na_3_PO_4_-coated NCO] and [Na|SPE|10 wt% NZSP-coated NCO] cells (a), cycle number dependences of the discharge capacity and coulombic efficiency (b), and internal resistance indexes (*R*) calculated from the charge–discharge voltages (c).

The charge–discharge profiles of the [Na|SPE|NCO] cells at 333 K are shown in Fig. 2 ((a) non-coated, (b) 5 wt% Na_3_PO_4_, (c) 10 wt% Na_3_PO_4_ and (d) 10 wt% NZSP coated NCO positive electrode). Na-based batteries using conventional NCO as the positive electrode active material typically exhibit multistep voltage plateaus owing to changes in the crystal structure of NCO during the charging and discharging processes.^34^ In this study, the cell with a non-coated positive electrode (a) exhibited a lower initial discharge capacity of *ca.* 36 mA h g^−1^ compared with the theoretical capacity (117 mA h g^−1^), and significant polarization derived to insufficient charge/discharge reactions without multistep plateaus. In addition, the cell with 5 wt% Na_3_PO_4_ (b) did not show a significant change in initial discharge capacity compared with the non-coated system (a), and a slight multistep plateau was observed in the first cycle. In contrast, the 10 wt% Na_3_PO_4_ (c) and 10 wt% NZSP (d), the higher initial discharge capacities of approximately 66 and 60 mA h g^−1^, respectively, compared with that of non-coated cells, and the suppressed overvoltage and polarization were indicated by a reduction in the internal resistance and the appearance of distinct multistep plateaus. As the reason for these reduction in internal resistance and improvements of initial discharge capacity, we proposed an interfacial protection model that involves the formation of a steady Na^+^ conduction pathway between active materials and at the NCO/SPE interface owing to partially and rigidly coated inorganic oxide materials onto the NCO particle surface. However, the cells using non-coated positive electrodes exhibited decreasing discharge capacities with cycling, whereas cycle degradation was suppressed in cells with Na_3_PO_4_- and NZSP-coated positive electrodes. In the case of LCO, which is similar to NCO, capacity degradation was attributed to the collapse of the crystal structure and the formation of electrochemically inert Co_3_O_4_.^27^ Thus, surface coatings composed of Na^+^-conducting inorganic oxides, such as Na_3_PO_4_ and NZSP, are expected to protect the NCO crystal structure and suppress Co_3_O_4_ formation, improving not only the capacity but also the degradation suppression in ASSBs using SPEs. The charge–discharge profiles for the 1st and 6th cycles of [Na|SPE|NCO] cells with 10 wt% Na_3_PO_4_ and 10 wt% NZSP are presented in Fig. 3(a). The profiles are similar in the voltage regions of each plateau derived from the multiple crystal structure changes in NCO, even though the capacities of the 1st cycle are slightly different for the two cells.^34^ Therefore, the inorganic oxide-coated positive electrodes underwent electrode reactions without inhibiting the intrinsic crystalline structural changes in NCO during the charge–discharge processes. Furthermore, in the 6th cycle, despite a decrease in the charge–discharge capacities compared with those of the 1st cycle, it was expected that 10 wt% NZSP exhibited greater degradation suppression than 10 wt% Na_3_PO_4_, owing to the reduction in polarization resistance and higher average discharge voltage.

Based on the results of the constant-current charge–discharge tests, the cycle dependences of the coulombic efficiencies and resistance index for internal cells (*R*) were calculated (see Fig. 3(b) and (c)). From Fig. 3(b), the discharge capacities and coulombic efficiencies of cells using positive electrodes coated with inorganic oxides (Na_3_PO_4_ and NZSP) were clear improved in almost all cycles compared with the non-coated positive electrode. Whereas the cells with non-coated positive electrodes exhibited significant capacity degradation until the 5th cycle, gradual degradation was observed in the 10 wt% Na_3_PO_4_ and 10 wt% NZSP cells. Therefore, the inorganic oxide coating on the surface of NCO particles was expected to help mitigate degradation, even with a small amount of coating (approximately 10 wt%). Furthermore, 10 wt% NZSP exhibited higher coulombic efficiency and smaller capacity degradation compared with 10 wt% Na_3_PO_4_, and is expected to effectively protect the NCO surface/crystal structure during charging/discharging processes. To investigate the resistance index, *R* was defined and calculated using eqn (1),

1 *R* = (*V*_charge_ − *V*_discharge_)/2*I*_operation_

where *I*_operation_ is the operating current value, and *V*_charge_ and *V*_discharge_ represent the average voltages of the charge and discharge processes, respectively. Although *R* increased with the increasing cycle number in all cell systems, indicating an increase in polarization and overvoltage during charge–discharge reactions, the 10 wt% Na_3_PO_4_ and 10 wt% NZSP exhibited a decrease in the increasing trends compared with the non-coated system. The degradation suppression effect of the inorganic oxide coating on the NCO surface was further confirmed from the viewpoint of internal resistance. In addition, concerning the increase in *R* values, 10 wt% Na_3_PO_4_ exhibited a drastic increase until the 9th cycle, whereas 10 wt% NZSP showed a gradual increase beyond the 15th cycle. The variations observed in coating materials are attributed to differences in coating morphology (particle size/particle shape) on the NCO surface or to ionic transport properties between the active material and electrolyte due to the differing in Na^+^ conductivity^35–37^ of NZSP and Na_3_PO_4_. However, 5 wt% Na_3_PO_4_ exhibited higher *R* values compared with the non-coated positive electrode after the 2nd cycle. This may be attributed to a significant increase in resistance resulting from current concentration and overcharging in areas with insufficient Na_3_PO_4_ coating during the initial cycle, due to the localized (insufficient) coating region onto the NCO surface. Therefore, the coatings suppressed degradation throughout the charge–discharge cycles in ASSBs using SPE, and 10 wt% NZSP the introduction of inorganic oxides onto the NCO surface was found to be crucial when applying NZSP provided the greatest improvement in performance by their high Na^+^ conductivity compared with Na_3_PO_4_.

### Morphology investigation of the inorganic oxide coatings

**Fig. 4:**
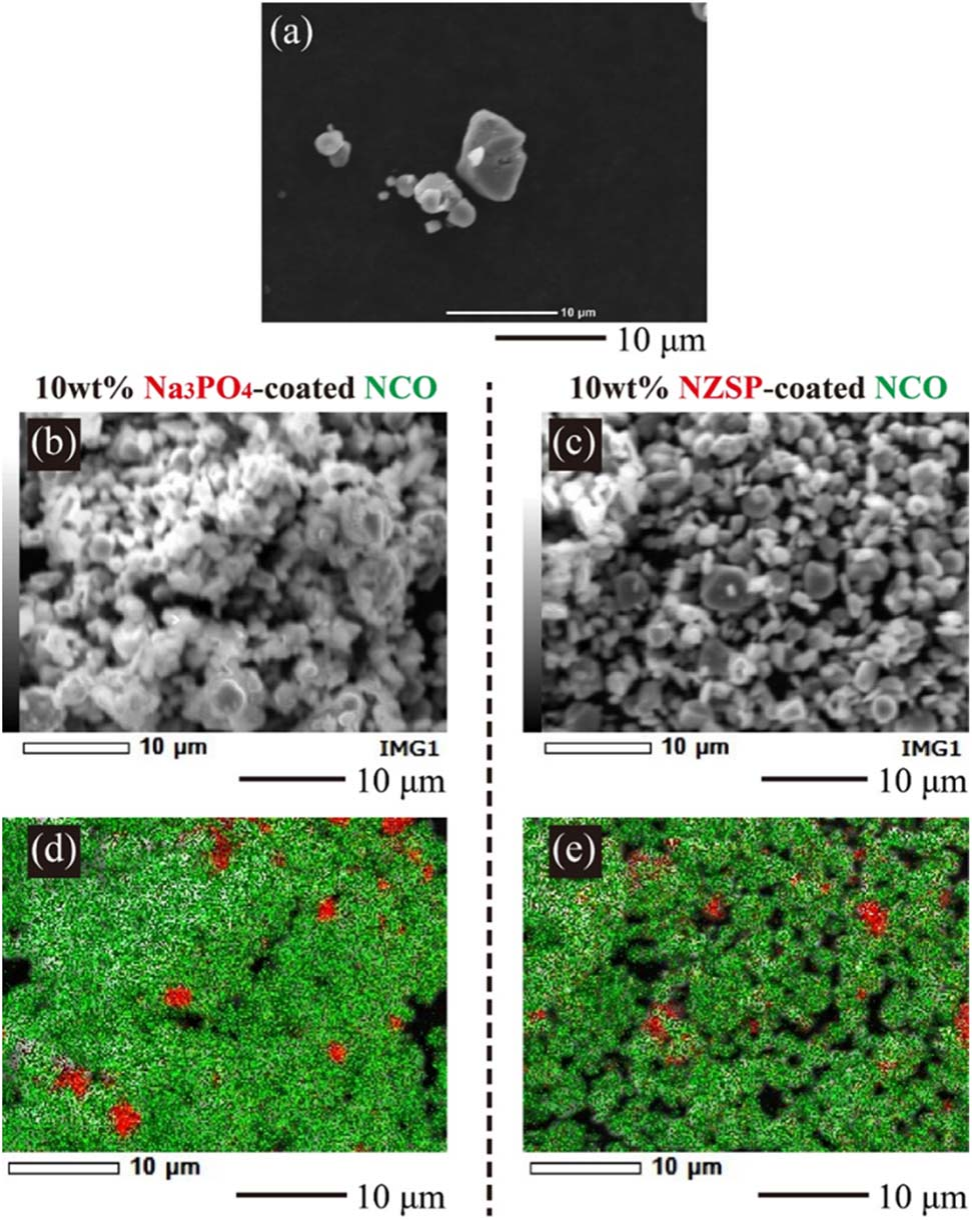
SEM and EDX mapping images of the NCO powder (SEM: (a)), 10 wt% Na_3_PO_4_-coated NCO powder (SEM: (b), EDX: (d)), and 10 wt% NZSP-coated NCO powder (SEM: (c), EDX: (e)).

To observe the coating morphology of inorganic oxides on NCO particles, SEM-EDX analysis was performed on the NCO, 10 wt% Na_3_PO_4_, and 10 wt% NZSP powders. SEM images of the NCO, 10 wt% Na_3_PO_4_, and 10 wt% NZSP powders are shown in Fig. 4(a)–(c), respectively, along with their elemental distributions presented in Fig. 4(d) and (e). NCO secondary particles were observed with sizes ranging from approximately 1 to 8 μm. The EDX mappings show Co (green) and P/Zr (red) for the NCO and inorganic oxides, respectively. The size of the primary NCO particles was approximately 2 to 3 μm after mixing, confirming a decrease in particle size. The small size of the primary NCO particles confirmed to help attenuate the formation of secondary particles or aggregates during the mixing process. From the EDX mapping (Fig. 4(d)), it was observed that Na_3_PO_4_ also had a smaller particle size after mixing compared to before mixing (approximately 2 μm, see Fig. S2†) and exhibited a local distribution near the NCO particles, as observed by the overlapped Co (green) and P (red) elements. In the NZSP system, there was only a slight change in particle size after the mixing process, but a local distribution near the NCO particles was also observed, similar to the case of Na_3_PO_4_. Therefore, the NCO powder containing Na_3_PO_4_ or NZSP after mixing suggested that the inorganic oxide was locally coated onto the NCO particle surface, even though they were partially isolated to some extent, while mostly maintaining the original particle size. From these results, it can be inferred that the aggregated structures collapsed during the mixing process, and the resulting pulverized powder adhered to the NCO particles, forming a coating on their surface. Additionally, the coating morphology of the NCO surface was similar, and no differences were observed in the particle size distributions between the 10 wt% Na_3_PO_4_ and 10 wt% NZSP. Therefore, the different changes in capacity and *R* with cycling (see Fig. 3(b) and (c)) were suggested to be caused by variations in the Na^+^ conductivity^35–37^ and electrochemical stability^36,38^ of NZSP and Na_3_PO_4_, rather than differences in coating morphology.

### Analysis of the degraded positive electrode in the [Na|SPE|NCO] cells

**Fig. 5:**
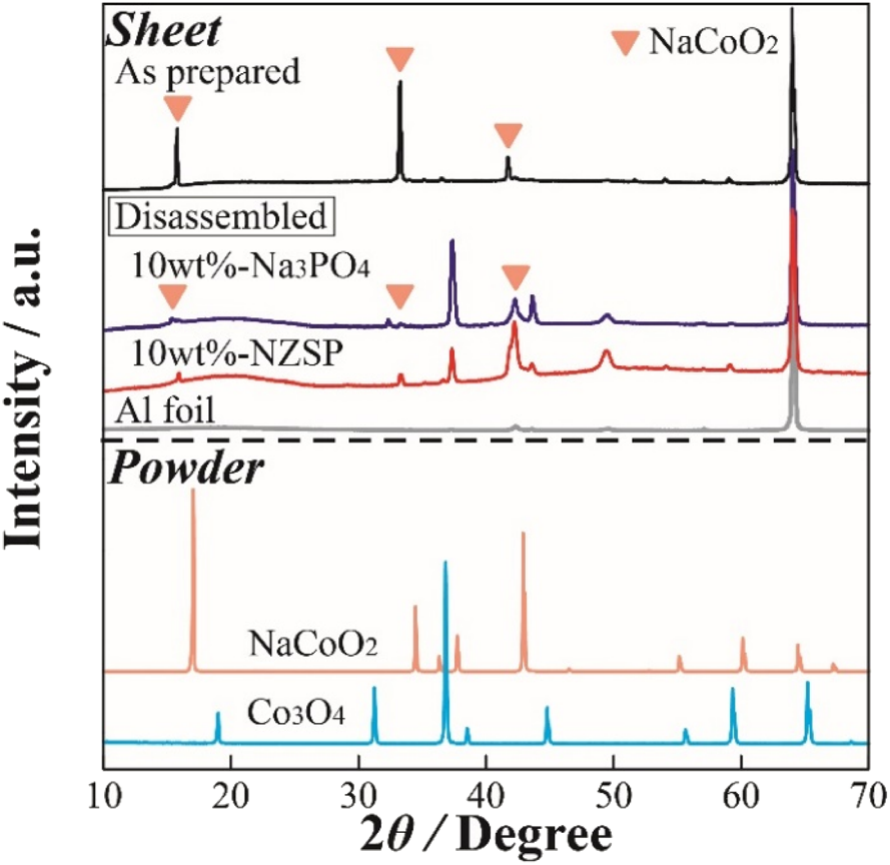
XRD patterns of the NCO electrode sheets in the as prepared and disassembled states, as well as the reference powder materials (NCO, Co_3_O_4_).

To observe the structural changes of NCO in the inorganic oxide-coated positive electrode after cycling, XRD measurements were performed on the positive electrode sheet obtained from the disassembled cell following the charge–discharge test. Fig. 5 displays XRD patterns of the non-coated positive electrode (as prepared), 10 wt% Na_3_PO_4_ (disassembled), 10 wt% NZSP (disassembled), Al foil (current collector), and NCO and Co_3_O_4_ powders. The XRD pattern of the NCO powder confirmed that it did not contain any impurity phases, exhibiting a crystal structure consistent with a single-phase O3-type layered oxide (ICSD: 96428). In addition, peaks corresponding to the (003), (006), and (104) planes of NCO were observed around 2*θ* = 16°, 33°, and 42° in the XRD pattern of the electrode sheet (as prepared) before charge–discharge, respectively. This observation suggests that there was no change or degradation in the crystal structure during the fabrication process of the composite positive electrode. Note that all composite positive electrode sheets exhibited a sharp diffraction peak around 65°, attributed to the Al foil (current collector), indicating the porosity and penetration depth of incident X-rays on the electrode surface. Moreover, in the disassembled positive electrode, although both 10 wt% Na_3_PO_4_ and 10 wt% NZSP exhibited main peaks derived from NCO before degradation through charge–discharge processes, all diffraction peaks, especially around 16° and 33°, indicated a change in crystal structure with the cycle number. Notably, a significant decrease in intensity and increase in peak width were observed compared with those of the as prepared sample. Therefore, in this battery system, the NCO partially maintains its crystal structure, facilitating the intercalation/deintercalation of Na^+^. This is supported by the confirmation of each diffraction peak derived from the active material in the disassembled electrode sheet, despite the occurrence of irreversible structural changes. Furthermore, diffraction peaks were observed around 37°, 44°, and 49° in the disassembled positive electrodes, which were not present in the as prepared positive electrode. These peaks can be attributed to the formation of Na^+^-deficient Na_0.64_CoO_2_ (ICSD: 246580), which was not intercalated during the discharge process after deintercalation in the charge process, or cobalt oxides, such as CoO_*x*_, intermediate in the collapsing structure of NCO. In particular, the Co element in the NCO crystal structure easily deteriorated because of reduction reactions with oxygen (ether oxygen) in the electrolyte during the charge–discharge process. These side products may accumulate at the NCO/SPE interface with cycling.

Based on these results, the degradation of [Na|NCO] cells with cycling and the mechanism of degradation suppression by the inorganic oxide coating were suggested as follows.

(1) Cell degradation occurs as the main crystal structure of NCO collapses, forming intermediate cobalt oxides, such as CoO_*x*_ and Co_3_O_4_, owing to the reduction of the CoO_2_ layer in the framework structure.

(2) Even though the generated CoO_*x*_ and Co_3_O_4_ inhibit the charge–discharge reaction of the cell because of their insulating properties, the capacity degradation with cycling is suppressed by preventing the formation of cobalt oxide. This was achieved through the formation of a steady coating layer of inorganic oxide with high chemical stability and Na^+^ conductivity on the NCO surface.

### Evaluation of the chemical stability of NCO

**Fig. 6:**
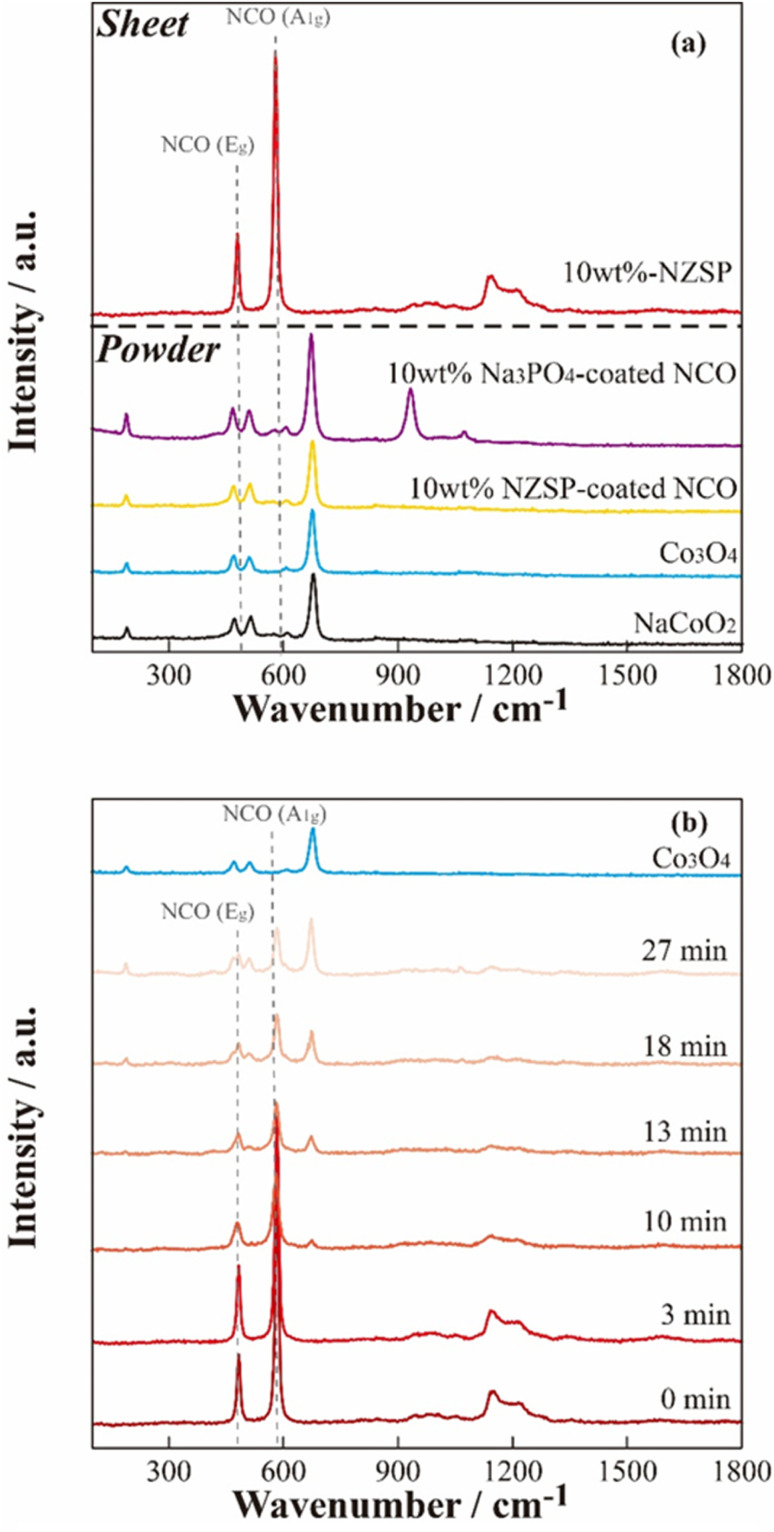
Raman spectra of the 10 wt% NZSP-coated NCO electrode sheet and the reference materials (a), and air exposure time dependence of the non-coated NCO electrode sheet (b).

To further investigate the microscopic structure of NCO surface, Raman spectroscopy was performed on each powder sample and positive electrode sheet under an Ar atmosphere. Fig. 6(a) displays Raman spectra of the NCO and Co_3_O_4_ powders, NCO powder mixed with inorganic oxides (10 wt% Na_3_PO_4_ and 10 wt% NZSP), and a 10 wt% NZSP positive electrode sheet. The peaks corresponding to E_g_ (twist vibration of O–Co–O) and A_1g_ (symmetric stretching vibration of Co–O) in layered oxides, such as LCO,^39^ were not observed in the NCO powder, and the Raman spectra were similar to those of Co_3_O_4_ powder (light blue). Furthermore, the wavenumbers of the peaks observed in the NCO powder closely agreed with the reported positions of vibrational modes corresponding to Co_3_O_4_ (194 cm^−1^ (F_2g_), 488 cm^−1^ (E_g_), 522 cm^−1^ (F_2g_), and 691 cm^−1^ (A_1g_)^40^). Therefore, the surface of the NCO powder can contribute to the collapse of the layered rock-salt structure and the formation of Co_3_O_4_ through side reactions with small amounts of moisture, even in an Ar atmosphere with a low dew point and low oxygen concentration. Thus, there are important technical issues related to its extreme sensitivity to the atmosphere. The spectra of inorganic oxide-coated NCO powders (10 wt% Na_3_PO_4_, 10 wt% NZSP) exhibited Co_3_O_4_ peaks similar to the non-coated NCO powder. These peaks were derived from Na_3_PO_4_ (ref. 41 and 42) at 930 and 1070 cm^−1^ in the case of 10 wt% Na_3_PO_4_. Conversely, the composite positive electrode sheet with 10 wt% NZSP clearly showed peaks at 490 and 590 cm^−1^, corresponding to E_g_ (twist vibration) and A_1g_ (symmetric stretching vibration) in the layered cobalt oxide,^39^ respectively. Therefore, despite the NCO in the powder state being sensitive to moisture and forming Co_3_O_4_ on the particle surface, these side products are effectively removed during the slurry preparation process, which involves the addition of the binder solution and stirring (see Fig. 1).

In addition, the time dependence of the Raman spectra for the non-coated positive electrode sheet was acquired when exposed to air to evaluate the chemical changes of NCO in the positive electrode sheet in the presence of moisture (Fig. 6(b)). The Raman spectra of pristine samples and those acquired immediately after exposure to air (∼3 min) exhibited peaks derived from E_g_ and A_1g_ in the NCO, suggesting that side products can be effectively removed by the positive electrode sheet preparation procedure, similar to the case of 10 wt% NZSP (see Fig. 6(a)). However, the intensity of the peaks derived from E_g_ and A_1g_ clearly decreased over time. Additionally, the Raman spectra after 10 min revealed new peaks corresponding to Co_3_O_4_, and these peaks significantly increased with time. This suggests that Co_3_O_4_ formed rapidly under atmospheric conditions, despite the prior removal of side products from NCO during the positive electrode sheet preparation process. Despite the chemical instability of the NCO active material, which easily collapses its layered structure and forms Co_3_O_4_ through side reactions with a small amount of moisture, the preparation process of the positive electrode sheet, specifically the addition of the binder and stirring, also contributed to suppressing the NCO degradation by removing the impurity on the particle surface.

**Fig. 7:**
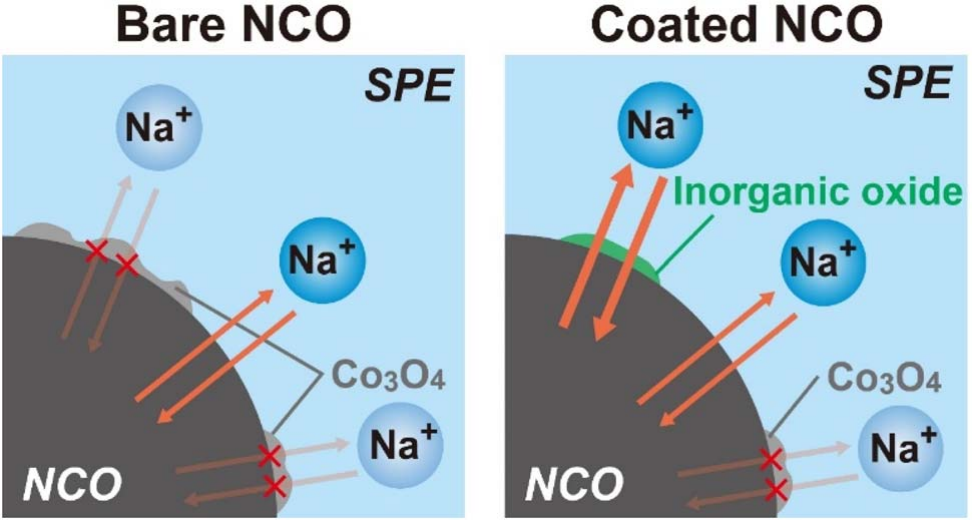
Schematic images of the proposed degradation behavior for non-coated and inorganic oxide-coated NCO interfaces.

According to the results obtained from the charge–discharge test, SEM-EDX, XRD, and Raman spectroscopy, schematic images depicting the proposed degradation behavior of NCO with cycling are shown in Fig. 7 (left: bare NCO, right: coated NCO). In both cell systems, reversible Na^+^ intercalation and deintercalation reactions take place at the NCO/SPE interface during the charge/discharge process. The bare NCO system was suggested to form insulators, such as CoO_*x*_ and Co_3_O_4_, on the NCO surface during the first cycle, leading to a significant decrease in electric capacity. This inhibits the Na^+^ intercalation and deintercalation reactions, reducing the reaction area of NCO. Therefore, the ASSB with non-coated NCO exhibits a degradation in capacity owing to the insulating and electrochemically inactive properties of the continuously formed Co_3_O_4_ (see Fig. 3(b)). Conversely, the coated NCO system exhibits a more extensive formation of the sufficient interfacial region and reduced Co_3_O_4_ formation compared with the non-coated system. This is attributed to the partial coating of the NCO particle surface by Na^+^-conducting inorganic oxides (see Fig. 4(d) and (e)). These coated regions appeared to facilitate the reversible Na^+^ intercalation/deintercalation reactions *via* Na^+^-conductive Na_3_PO_4_ and NZSP, suppressing capacity degradation with cycling and avoiding a reduction in the reaction area. In addition, the chemical stability of the inorganic oxides (Na_3_PO_4_, NZSP) may have contributed to the improved cycling performance by suppressing the formation of Co_3_O_4_ and side reactions with SPE. In addition to suppressing capacity degradation, the coatings enable the application of active materials with low chemical stability in the preparation of positive electrode sheets for Na-based batteries. Therefore, this study demonstrates that active materials with low electrochemical/chemical stabilities can find desirable applications in various batteries, including Na-based system by coating of inorganic oxides. *Operando* Raman measurements and AC impedance analysis^43^ will also be conducted to analyze the protective effect of inorganic electrolytes. In the future, further suppression of the degradation is anticipated by achieving a uniform coating on the surface of NCO and advanced positive electrode^44^ particles, either by increasing the amount of inorganic oxide or applying various techniques such as mechanical mixing (ball milling) and spray coating.

## Conclusions

All-solid-state Na polymer batteries ([Na|SPE|NCO] cells) were fabricated using Na_3_PO_4_- or NZSP-coated NCO as the positive electrode active material, and were evaluated by conducting constant-current charge–discharge tests. Morphological changes and the mechanism for suppressing degradation in the batteries were investigated by examining the structures and surface morphologies of the positive electrode sheets before and after cycling. The inorganic oxide coating increased the initial discharge capacity from approximately 36 to 60 mA h g^−1^, and improvements were also observed in the cycle performance and coulombic efficiency. In addition, the increase in the internal resistance index of the cells was suppressed, revealing that the suppression of degradation was more effective with NZSP than with Na_3_PO_4_. Based on EDX mapping, the similar coating morphology was observed for the inorganic oxides, and differences in the performance were attributed to the Na^+^ conductivity and electrochemical stability of each inorganic oxide. Moreover, the layered structure of NCO collapsed and formed byproducts (*e.g.*, CoO_*x*_) with cycling, and the capacity degradation was suggested to increase owing to their insulating and electrochemical properties. In contrast, the coated NCO was protected from degradation, and the inorganic oxides formed a conduction pathway for reversible Na^+^ intercalation/deintercalation. Owing to their high electrochemical stability, the coating layer provided a stable interfacial region where byproducts like CoO_*x*_ cannot easily formed due to the high chemical stability of coated inorganic oxides. Finally, the chemically unstable NCO powder tends to undergo side reactions with small amounts of moisture or other contaminants, and the addition of the binder and stirring during the fabrication process of positive electrode sheets contributed to the protection of the NCO surface in an atmosphere. Overall, the inorganic coatings contribute to suppress the degradation of the positive electrode/electrolyte interface and support the application of active materials with low chemical stability in all-solid-state batteries.

## Data availability

The data supporting this article have been included as part of the ESI.†

## Conflicts of interest

There are no conflicts to declare.

## Supplementary Material

RA-014-D4RA02957G-s001
