# Supplementary material for: Interfacial modification of NaCoO2 positive electrodes with inorganic oxides by simple mixing and the effects on all-solid-state Na batteries
Source: RSC Adv. 2024 Jun 19;14(28):19726–34. doi: 10.1039/d4ra02957g (PMC11188668; doi:10.1039/d4ra02957g)
Supplement: RA-014-D4RA02957G-s001 [file RA-014-D4RA02957G-s001.pdf]

Electronic Supplementary Material (ESI) for RSC Advances.
This journal is © The Royal Society of Chemistry 2024

Electric Supplementary Information

Interfacial Modification of $NaCoO_2$ Positive Electrodes with Inorganic Oxides by Simple Mixing and the Effects on All-Solid-State Na Batteries

Takaaki Ichikawa, Koji Hiraoka and Shiro Seki*

Graduate School of Applied Chemistry and Chemical Engineering, Kogakuin University, 2665-1 Nakano-machi, Hachioji-shi, Tokyo 192-0015, Japan.

---

* Corresponding author. Tel: +81-42-628-4568; fax: +81-42-628-4568.

E-mail: shiro-seki@cc.kogakuin.ac.jp (S. Seki)

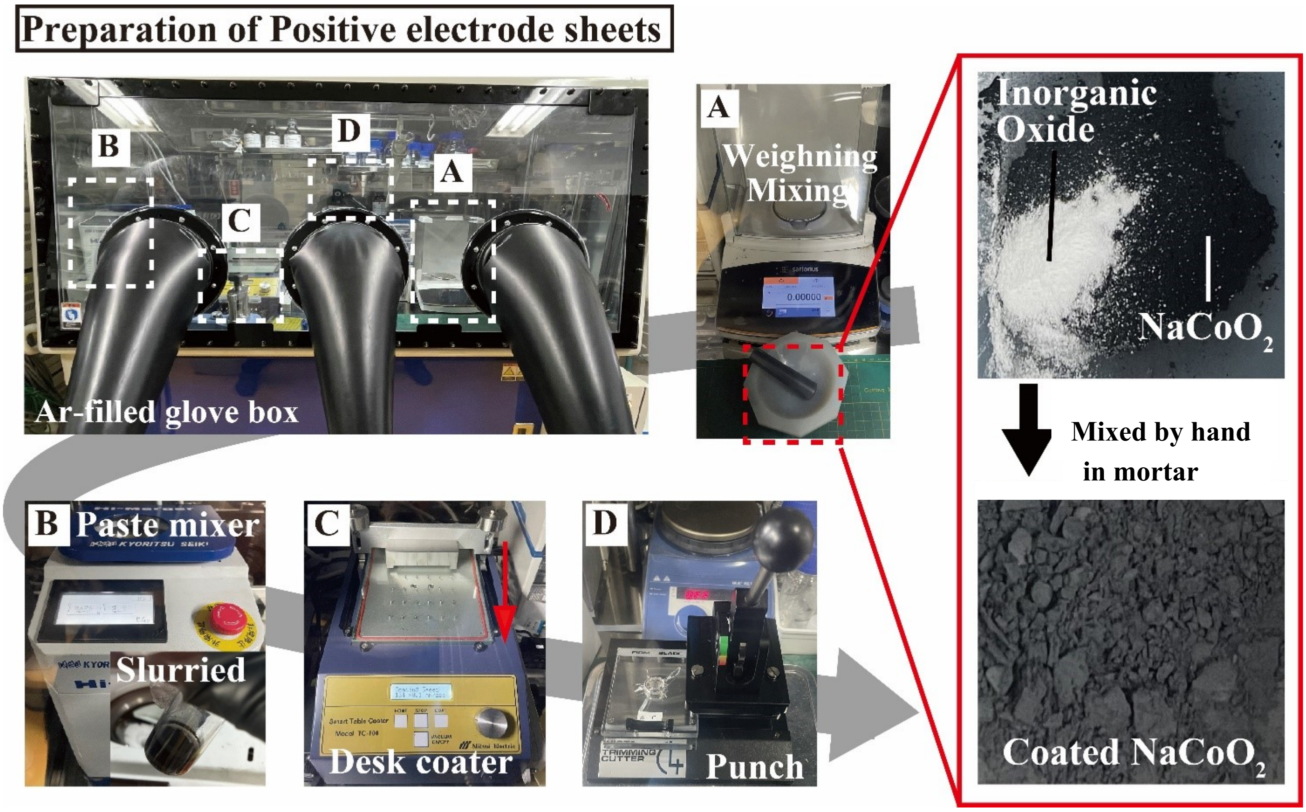

Figure S-1. Preparation scheme of inorganic oxide-coated positive electrode sheets for all-solid-state Na polymer batteries.

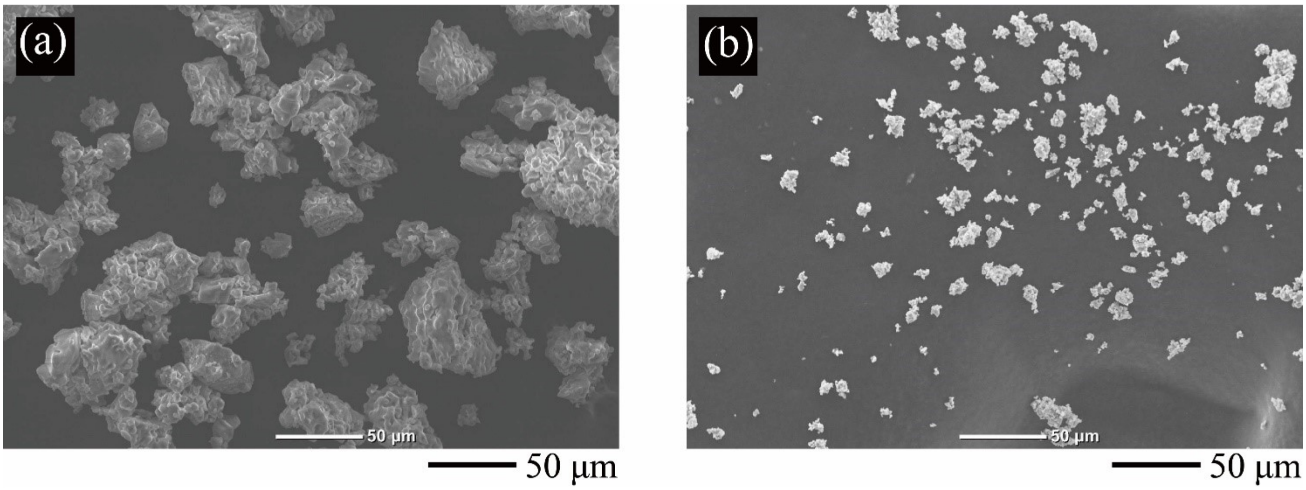

Figure S-2. SEM images of Na_3PO_4 powder (a) and NZSP- powder (b) before mixing, respectively.
